# After the Battle

**Published:** 1887-06-25

**Authors:** 


### After the Battle.

Nearly a millennium ago Peter the Hermit roused all Europe—princes, knights, and people—to enter upon a crusade for the deliverance of the holy sepulchre from the hands of the Infidels. It would be impossible to arouse either princes, knights, or people for any such purpose now. In those days ideas held the ground, and for an idea a man encountered danger on the battle-field, and if necessary, death at the stake. We think we are less visionary and more practical in these times. Perhaps we are, but we do not always take up the practical duties approved by our judgment with the disinterested enthusiasm which characterised the crusaders of the Plantagenet period. It is better and more Christian to try to save men's lives by art and skill than to destroy them on the battle-field ; but a soldier who fights and kills his fellow-men by the score may be intrinsically a much nobler being than a physician or a surgeon whose whole force and skill are given up to the saving of life. So far as the merit of the individual actor is concerned, everything depends upon the motive and the energy of enthusiasm with which the end is pursued.

The object of the religious crusades of the Angevin kings was visionary and of little value in itself. The inspiring motives of the crusaders and the chivalrous valour of their lives were often worthy of the sincerest admiration. It is in the want of enthusiasm and self-abandonment that modern men fail of nobility. Our heads are moderately clear on a number of important questions, but our hearts in many instances are miserably and meanly cold.

In the latter-day "crusade" of which we write there has been no want of ardour among the knights and leaders. Princes of the blood have vied with princes of the Church, and the free-lances of religion and politics have borne themselves in the fray with as much zeal and courage as their more orthodox brethren. But there has been a cold and meagre response among the men and women of the rank and file. Time was when if a Peter the Hermit could inspire the natural leaders of the people, the people themselves caught the contagion of enthusiasm and became more fervid than their leaders. In the case of the "crusade" against indifference this rule has been reversed. There have not been wanting Hermit Peters who have given themselves body and soul, heart and life to the movement. Not to mention others, the veteran Sir Sydney Waterlow, and the almost equally experienced Sir Edmund Currie, with a host of others, have striven with an ardour which nothing could quench, and a persistence which has known no limits, to create and extend among all classes a deep, intelligent, and permanent sympathy with voluntary hospitals and their great and unselfish work. Up to he present time the results have not been equal to the efforts put forth. It is true that on all sides we hear of additions to the Hospital Sunday collections, which will make the total sum probably much greater than it has ever been before. But that was only a part of the object for which the crusade was engaged in. We do not want to employ the vast machinery of which princes of the blood, premiers, archbishops, statesmen of all ranks, free-lances of all faiths form constituent parts, for the comparatively poor purpose of raising fifty instead of forty thousand pounds for the Sunday Fund. Even if the amount collected reach the larger figure it is still only half of what is needed, and ought easily to be raised But the object of the crusade was much more significant than that of mere money-raising. It was, as we have said, nothing less than the conversion of all the indifferent public into active, intelligent, and sympathetic supporters of hospitals.

To a certain limited extent the crusade has been successful. In a larger and profounder sense we cannot claim for it an overwhelming success. Now there are some Hermit Peters connected with the hospital world who are fully resolved that sooner or later the crusade shall result in a series of victories which shall carry all before them. That being so, and there being no idea of despair, but a kind of Wellingtonian stubbornness which only sees in temporary checks fresh reasons for new consideration, new methods, and new and more resolute efforts towards final victory, we have to ask the friends of the Fund to consider—to consider seriously and intelligently—why it was that the meetings during the Hospitals Fortnight were so poorly attended, and why the dense clouds of indifference steadily refused to be rolled away, in spite of the shining of so many religious, political, philanthropic, and social luminaries of the very first order of brilliance and heat-giving power ? There are the facts that the meetings were poor and the enthusiasm feeble. What was the reason, or reasons?

There are two possible classes of reasons. In the first place there may be a want of sympathy with the object of the crusade, and with hospitals and their work ; or secondly, there may be a vast amount of ignorance on the subject of medical charities : ignorance of the extent and variety of their operations : of the magnitude of their necessary expenditure, and of the comparative smallness of their accumulated resources. Is it want of sympathy which is the cause of the public indifference, or is it ignorance? There can be no kind of doubt as to what is the answer to the question. Sympathy with hospitals, in this country, is universal ; from the illustrious Sovereign whose Jubilee has just been celebrated, to the poorest street *gamin* who has seen the outside of the hospital walls, there is hardly an exception to this universal feeling. Ignorance, then, and ignorance only, is the real cause of that neglect of hospitals which prevails among so considerable a proportion of all classes of the people. This ignorance cannot be wondered at. What means have been employed to arouse the public interest and to enlighten the public mind? Practically no means at all. In trade, where an industry must keep itself before the public in order to keep itself in existence, publicity, knowledge, enlightenment of the people is as the very breath of life. Until a few months ago hospitals as a whole had no means whatever of saying one single collective word to the million-eared public which wishes to be informed about them, and which possesses an unlimited power of giving them support. Whilst every little trade and diminutive industry has had its Journal, its living tongue, proclaiming on the housetop everything about its constituency which the world could by any possibility desire to know, the huge hospital system has floundered about like a half-starved leviathan, and made no effort to beg, borrow, steal, or develop a tongue whereby it could proclaim to all the world that it was very hungry, and that, since it was a monster which existed solely for the well-being of all mankind, the world had better feed it, and make some effort to keep it alive and well. If the hospital system had possessed such a tongue during the last two decades, and had used it with intelligence and persistence, we make bold to say that the "Crusade against Indifference," which has just been temporarily suspended, would never have needed to be entered upon at all.

The conclusion of the whole matter is this: the public must be educated, informed, enlightened. The country is well able to support its voluntary hospitals; it is as willing as it is able; it will advantage itself in every kind of way by supporting them; it will make the Victorian Era great and famous in this as it is in other directions by supporting them. If the hospitals are not supported it will not be the fault of the public, but of hospital managers themselves, who either "cannot discern the signs of the times," or discerning them, are not capable of guiding their ship into the deep and smooth waters of public approval and financial prosperity.

